# Supplementary material for: Dynalign II: common secondary structure prediction for RNA homologs with domain insertions
Source: Nucleic Acids Res. 2014 Nov 21;42(22):13939–48. doi: 10.1093/nar/gku1172 (PMC4267632; doi:10.1093/nar/gku1172)
Supplement: SUPPLEMENTARY DATA [file supp_gku1172_nar-02021-z-2014-File012.zip › manual/RNA_class/html/functions_func_0x67.html]

RNAstructure Classes: Class Members - Functions

- Main Page
- Classes
- Files

- Class List
- Class Hierarchy
- Class Members

- All
- Functions
- Variables
- Typedefs

- a
- b
- c
- d
- e
- f
- g
- h
- m
- n
- o
- p
- r
- s
- t
- v
- w
- ~

### - g -

- GenerateAllSuboptimalStructures()
  : RNA- get\_dGIndex()
    : Multilign\_object- get\_energies()
      : Multilign\_object- get\_gap()
        : Multifind\_object- get\_pair\_alignments()
          : Multilign\_object- GetBackboneType()
            : RNA- GetBestPairEnergy()
              : Dynalign\_object- GetBreakTargetDG()
                : Oligowalk\_object- GetCommentString()
                  : RNA- GetDat()
                    : Thermodynamics- GetDatatable()
                      : Thermodynamics- GetDuplexDG()
                        : Oligowalk\_object- GetEnergyRead()
                          : Thermodynamics- GetEnsembleEnergy()
                            : RNA- GetEnthalpyTable()
                              : Thermodynamics- GetErrorCode()
                                : HybridRNA
                                , Multilign\_object
                                , RNA
                                , TwoRNA- GetErrorMessage()
                                  : Dynalign\_object
                                  , HybridRNA
                                  , Multilign\_object
                                  , Oligowalk\_object
                                  , RNA
                                  , TwoRNA- GetErrorMessageString()
                                    : RNA
                                    , TwoRNA- GetForbidIntramolecular()
                                      : HybridRNA- GetForcedAlignment()
                                        : Dynalign\_object- GetForcedDoubleStranded()
                                          : RNA- GetForcedFMNCleavage()
                                            : RNA- GetForcedModification()
                                              : RNA- GetForcedPair()
                                                : RNA- GetForcedProhibitedPair()
                                                  : RNA- GetForcedSingleStranded()
                                                    : RNA- GetFreeEnergy()
                                                      : RNA- GetIndexSeq()
                                                        : Multilign\_object- GetInputFilenames()
                                                          : Multilign\_object- GetIterations()
                                                            : Multilign\_object- GetLabelXCoordinate()
                                                              : RNA- GetLabelYCoordinate()
                                                                : RNA- GetLowestEnergy()
                                                                  : Dynalign\_object- GetMaxDsv()
                                                                    : Multilign\_object- GetMaximumPairingDistance()
                                                                      : RNA- GetMaxPairs()
                                                                        : Multilign\_object- GetNucleotide()
                                                                          : RNA- GetNucleotideXCoordinate()
                                                                            : RNA- GetNucleotideYCoordinate()
                                                                              : RNA- GetNucType()
                                                                                : Multilign\_object- GetNumberOfForcedDoubleStranded()
                                                                                  : RNA- GetNumberOfForcedFMNCleavages()
                                                                                    : RNA- GetNumberOfForcedModifications()
                                                                                      : RNA- GetNumberOfForcedPairs()
                                                                                        : RNA- GetNumberOfForcedProhibitedPairs()
                                                                                          : RNA- GetNumberOfForcedSingleStranded()
                                                                                            : RNA- GetOligoOligoDG()
                                                                                              : Oligowalk\_object- GetOligoSelfDG()
                                                                                                : Oligowalk\_object- GetOverallDG()
                                                                                                  : Oligowalk\_object- GetPair()
                                                                                                    : RNA- GetPairEnergy()
                                                                                                      : RNA- GetPairProbability()
                                                                                                        : RNA- GetPairs()
                                                                                                          : Multilign\_object- GetProgress()
                                                                                                            : RNA
                                                                                                            , Multilign\_object- GetRNA1()
                                                                                                              : TwoRNA
                                                                                                              , HybridRNA- GetRNA2()
                                                                                                                : TwoRNA
                                                                                                                , HybridRNA- GetSequenceLength()
                                                                                                                  : RNA- GetSequenceNumber()
                                                                                                                    : Multilign\_object- GetSHAPEIntercept()
                                                                                                                      : Multilign\_object- GetSHAPESlope()
                                                                                                                        : Multilign\_object- GetStructure()
                                                                                                                          : RNA- GetStructureNumber()
                                                                                                                            : RNA- GetTemperature()
                                                                                                                              : Thermodynamics
                                                                                                                              , TwoRNA
                                                                                                                              , Multilign\_object- GetTm()
                                                                                                                                : Oligowalk\_object

---

Generated on Tue Jul 30 10:52:29 2013 for RNAstructure Classes by 
 1.5.7.1
